# Exploring the Multifaceted Phytochemical Profile of *Nigella sativa* and the Therapeutic Potential of Thymoquinone

**DOI:** 10.3390/ph19030503

**Published:** 2026-03-18

**Authors:** Mohamed A. Fareid, Gamal M. El-Sherbiny, Nancy M. Elafandy, Nagat E. Eltoum, Mohamed S. Othman, Mohamed Shawky, Ahmad S. El-Hawary, Fatma A. Hamada, Amira Salah El-Din Youssef

**Affiliations:** 1 Clinical Laboratory Science Department, Applied Medical Science College, University of Ha’il, Hail 2440, Saudi Arabia; 2 Botany and Microbiology Department, Faculty of Science, Al-Azhar University, Cairo 11884, Egypt; 3 Clinical Nutrition Department, Applied Medical Science College, University of Ha’il, Hail 2440, Saudi Arabia; 4 Biochemistry Department, College of Medicine, University of Ha’il, Hail 2440, Saudi Arabia; 5 Basic Sciences Department, First Year of Health and Medical Colleges, University of Ha’il, Hail 2440, Saudi Arabia; 6 Virology and Immunology Unit, Cancer Biology Department, National Cancer Institute, Cairo University, Cairo 11796, Egypt

**Keywords:** *N. sativa*, thymoquinone, UHPLC/QTOF-MS, TLC, RP-HPLC, antibacterial, biofilm inhibition, antioxidant, anti-inflammatory, antidiabetic activity

## Abstract

**Background:** *Nigella sativa* (black cumin) seeds are renowned for their ethnomedicinal significance and are rich in bioactive phytochemicals, which contribute to food preservation and the prevention of various diseases through their antimicrobial and antioxidant properties. Accordingly, this study aimed to characterize the phytochemical composition of *N. sativa* seed extracts, isolate thymoquinone, and assess their antibacterial, antibiofilm, antioxidant, anti-inflammatory and antidiabetic activities. **Methods:** *Nigella sativa* seed extracts were prepared using solvents of increasing polarity and analyzed for phytochemical content. Metabolite profiling was performed using UHPLC/QTOF-MS. Thymoquinone, the major constituent, was isolated via thin-layer chromatography (TLC), further purified using semi-preparative reverse-phase high-performance liquid chromatography (RP-HPLC), and evaluated in vitro for antibacterial, antibiofilm, antioxidant, anti-inflammatory, and antidiabetic activities. **Results:** Extraction yields ranged from 5.5% to 8.4% (*w*/*w*), with methanol yielding the highest phenol (6.34 ± 0.31 mg GAE/mL) and flavonoid (5.12 ± 0.26 mg QE/mL) contents. UHPLC/QTOF-MS revealed a chemically diverse profile dominated by thymoquinone (58% relative abundance), alongside *p*-cymene, carvacrol, longifolene, and nigellidine. Thymoquinone (Rf = 0.56) was initially isolated from the methanolic extract with a yield of 270 mg/g and further purified from preparative TLC fractions using semi-preparative RP-HPLC, affording 82 mg of >95% pure compound with a 68.3% recovery, suitable for subsequent biological assays. It inhibited Gram-positive and Gram-negative bacteria, with MICs of 62.5 µg/mL against *Staphylococcus aureus*, *Bacillus subtilis*, and *Listeria monocytogenes*; 125–250 µg/mL against *Escherichia coli* and *Salmonella typhimurium*; and 500 µg/mL against *Pseudomonas aeruginosa*. Thymoquinone reduced biofilm formation (>80% at 25–50 µg/mL; MBIC_50_ ≈ 5.4–11.6 µg/mL), exhibited antioxidant activity (DPPH IC_50_ = 52.3 ± 2. 1 µg/mL; ABTS IC_50_ = 41.6 ± 1.9 µg/mL), stabilized erythrocyte membranes (IC_50_ ≈ 14.8 µg/mL), and inhibited carbohydrate-hydrolyzing enzymes, with stronger inhibition of α-glucosidase (~92%) than α-amylase (~84%) at 128 µg/mL. **Conclusions:** Thymoquinone is a major bioactive constituent of *N. sativa* seeds, exhibiting consistent multi-target in vitro activity. These findings highlight its functional relevance and in vivo investigations to establish therapeutic potential.

## 1. Introduction

Medicinal plants remain a vital source of bioactive compounds for the prevention and management of human diseases, particularly in the face of escalating antimicrobial resistance, chronic inflammatory conditions, oxidative stress-related disorders, and metabolic diseases such as diabetes mellitus. Despite significant advances in synthetic drug discovery, the demand for safer, multi-target, and naturally derived therapeutic agents continues to grow worldwide [1]. Within this context, *N. sativa* L. (*Ranunculaceae*), commonly known as black cumin, has attracted increasing scientific attention due to its long-standing use in traditional medicine and its wide range of reported pharmacological properties [2]. The plant is native to the Eastern Mediterranean, North Africa, Southeast Asia, and the Indian subcontinent and is now widely cultivated across regions including Greece, Turkey, Egypt, Iran, Saudi Arabia, India, and Pakistan [3,4,5,6].

Traditionally, *N. sativa* seeds have been employed in the treatment of respiratory, gastrointestinal, inflammatory, and metabolic disorders across multiple traditional medical systems, including Unani, Ayurvedic, Siddha, and Middle Eastern folk medicine [5,7,8,9,10]. Phytochemical investigations have revealed that the seeds contain a complex mixture of volatile and non-volatile constituents, among which thymoquinone is widely recognized as the principal bioactive compound responsible for many of the plant’s therapeutic effects [2,7,8]. In addition to thymoquinone, other constituents such as *p*-cymene, α-thujene, longifolene, β-pinene, α-pinene, and carvacrol have been identified and are believed to contribute synergistically to the plant’s pharmacological profile. Consequently, *N. sativa* has been reported to exhibit anti-inflammatory, antioxidant, antibacterial, antidiabetic, and diuretic activities and is commercially formulated into essential oils, powders, capsules, and standardized extracts for medicinal use [5,10,11,12].

Effect-directed fractionation has emerged as a powerful strategy for isolating pharmacologically active constituents from medicinal plants by directly coupling chemical separation with bioactivity evaluation [13]. Integrative network pharmacology frameworks that incorporate microbial and hepatic biotransformation have further advanced mechanistic understanding of complex herbal bioactive, particularly in neurological and inflammatory diseases [14]. Mechanism-focused analyses of individual natural compounds demonstrate that multi-target modulation is a defining feature underlying the therapeutic potential of plant-derived molecules in nervous system disorders [15]. High-resolution analytical techniques, including HPLC/QTOF-MS combined with molecular networking, now enable comprehensive chemical profiling of complex herbal formulations and facilitate confident annotation of bioactive constituents [16]. Detailed pharmacological investigations of isolated phytochemicals reveal that structure–activity relationships critically govern anticancer and anti-inflammatory efficacy [17]. The availability of genome-scale sequencing and annotation resources has strengthened links between biosynthetic capacity, chemical diversity, and functional bioactivity in natural product research [18]. Immunopharmacological studies of traditional medicinal plants further indicate that therapeutic efficacy often arises from coordinated modulation of inflammatory and immune pathways rather than single-target effects [19]. Emerging evidence also shows that plant-derived oils and metabolites can reverse inflammation-associated senescence and prevent disease relapse through metabolic reprogramming [20,21].

Although numerous studies have documented the antibacterial, antioxidant, anti-inflammatory, and antidiabetic activities of *N. sativa* extracts or isolated thymoquinone [2,4,5,6,7,8,9,10,11,12,13,14,15,16,17,18,19,20,21,22,23], most of these investigations remain limited in scope. Many rely on crude extracts without comprehensive chemical characterization, focus on a single biological activity, or lack mechanistic integration and rigorous statistical validation.

A major gap in literature is the lack of studies integrating comprehensive phytochemical profiling, targeted thymoquinone purification, and systematic evaluation of its multifunctional biological activities using standardized and statistically robust methodologies. In particular, its antidiabetic potential—especially the selective inhibition of α-glucosidase over α-amylase—remains insufficiently characterized. Similarly, its antibiofilm efficacy at sub-inhibitory concentrations, a critical factor in mitigating persistent and foodborne bacterial infections, has not been comprehensively investigated [24,25,26,27,28].

The present study was therefore designed to address these gaps using an integrated analytical and biological approach, including comprehensive chemical characterization of *N. sativa* seed extracts by UHPLC/QTOF-MS, targeted purification and confirmation of thymoquinone, and systematic evaluation of its antibacterial, antibiofilm, antioxidant, anti-inflammatory, and antidiabetic activities. Importantly, this study directly correlates phytochemical profiling with functional bioactivity and incorporates rigorous statistical comparisons with standard reference drugs.

## 2. Results

### 2.1. Phytochemical Composition of N. sativa Seed Extracts

The extraction of *N. sativa* powdered seeds (100 g) was performed sequentially using solvents of increasing polarity-hexane, ethyl acetate, methanol, and ethanol (500 mL each, 24 h, room temperature)-yielding 5.5%, 6.2%, 8.4%, and 7.2% (*w*/*w*), respectively. Solvent polarity significantly influenced extraction efficiency, with polar solvents yielding higher amounts of most phytochemical classes. The methanolic extract contained the highest concentrations of phenols (6.34 ± 0.31 mg GAE/mL), flavonoids (5.12 ± 0.26 mg QE/mL), alkaloids (4.21 ± 0.24 mg/mL), and saponins (3.96 ± 0.21 mg/mL), followed closely by the ethanolic extract, as shown in Table 1. In contrast, the hexane extract contained significantly lower amounts of polar phytochemicals, with saponins and tannins undetectable, reflecting the limited ability of non-polar solvents to extract hydrophilic compounds. The ethyl acetate extract demonstrated moderate efficiency across most classes, particularly for phenols and flavonoids, indicating its suitability for semi-polar constituents. Lipophilic compounds such as steroids and terpenoids were more abundant in the hexane extract. Statistical analysis revealed significant differences (*p* < 0.05) among the extracts for all phytochemical classes, and post hoc least significant difference (LSD) tests confirmed that methanolic and ethanolic extracts contained significantly higher levels of phenols, flavonoids, alkaloids, and saponins compared to ethyl acetate and hexane extracts.

### 2.2. UHPLC/QTOF-MS Analysis of N. sativa Seed Methanolic Extract

**Figure 1:**
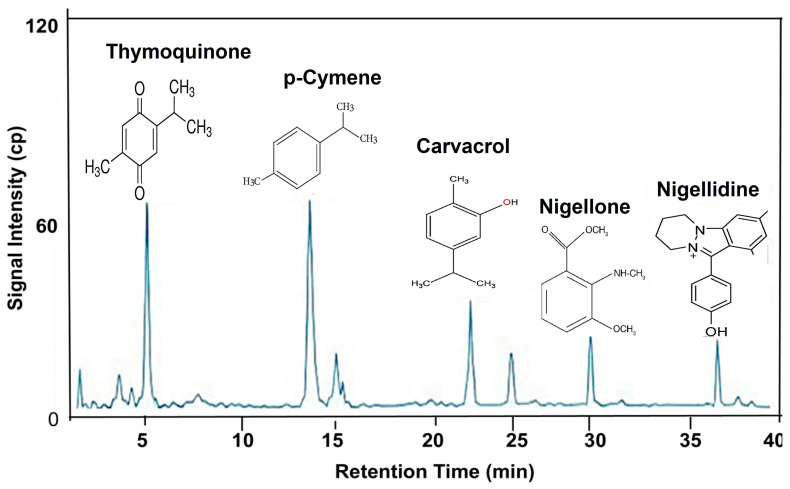
UHPLC-QTOF total ion chromatogram (TIC) of *N. sativa* methanolic seed extract in positive ion mode.

The chemical composition of the *N. sativa* seed methanolic extract was comprehensively characterized using UHPLC/QTOF-MS, enabling high-resolution separation and accurate mass identification of its phytochemical constituents. The total ion chromatogram (Figure 1) displayed a well-resolved profile with multiple peaks distributed across the chromatographic run, reflecting a chemically diverse extract. Thymoquinone was identified as the dominant constituent, appearing as a prominent early-eluting peak (Rt ≈ 5.21 min) and accounting for approximately 58% of the total ion chromatogram, supporting its classification as the major bioactive component of the extract. Additional compounds detected at lower relative abundances included *p*-cymene (Rt ≈ 14–15 min), carvacrol (Rt ≈ 21 min), nigellone (Rt ≈ 29.12 min), and nigellidine (Rt ≈ 37.32 min), each identified based on accurate mass measurements, characteristic fragmentation patterns, and comparison with database references (Table 2). The elution behavior of these metabolites reflects differences in molecular weight and polarity, with less polar terpenoids eluting later in the gradient. Together, these findings confirm that the methanolic extract of *N. sativa* is rich in quinones, terpenoids, and alkaloid-type compounds, with thymoquinone representing the most abundant MS feature.

### 2.3. Isolation and Purification of Thymoquinone from N. sativa Methanolic Extract

#### 2.3.1. Isolation and Purification by TLC

**Figure 2:**
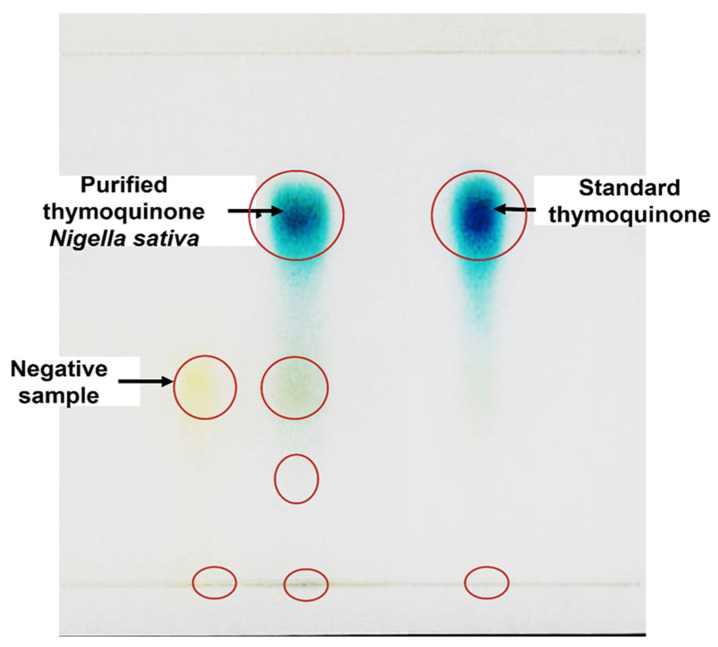
The TLC plate shows the separation of purified thymoquinone isolated from *N. sativa* (**middle lane**), a negative control sample (**left lane**), and standard thymoquinone (**right lane**). The purified extract exhibits a spot with similar color intensity and migration (Rf value) to the standard thymoquinone, confirming the presence of thymoquinone in the *N. sativa* extract, while no corresponding spot is observed in the negative sample.

Figure 2 illustrates the TLC fractionation of the *N. sativa* seed methanolic extract in comparison with an authentic thymoquinone standard. Chromatography was performed on silica gel plates using n-hexane: ethyl acetate (8:2, *v*/*v*) as the mobile phase. The developed chromatogram revealed a distinct, intense, and well-resolved band in the methanolic extract that co-migrated precisely with the thymoquinone reference standard, exhibiting an identical retention factor (Rf ≈ 0.56). The close agreement in Rf value, band intensity, morphology, and coloration strongly support the presence of thymoquinone as a major constituent of the methanolic extract. For isolation, the silica gel corresponding to the thymoquinone band was carefully scraped from the TLC plate and eluted with methanol. Repeated TLC runs using a total of 3.0 g of methanolic extract allowed effective enrichment and recovery of the target compound, yielding approximately 810 mg of thymoquinone (~27% recovery).

#### 2.3.2. Purification by RP-HPLC

Semi-preparative reversed-phase HPLC was employed to further purify thymoquinone obtained from preparative TLC. A total of 120 mg of the TLC-enriched fraction, presumed to contain thymoquinone as the major constituent, was subjected to repeated semi-preparative HPLC injections under optimized chromatographic conditions.

**Figure 3:**
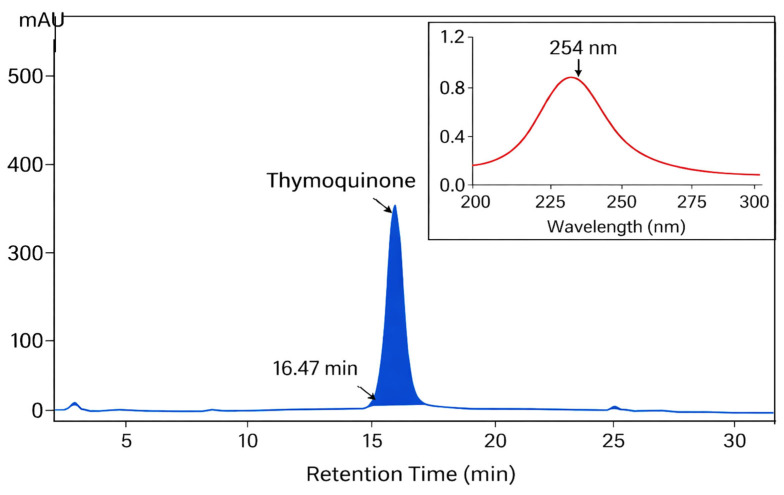
RP-HPLC chromatogram and UV spectrum of purified thymoquinone from *N. sativa*. Analytical RP-HPLC at 254 nm shows a single dominant peak at RT = 16.47 min (5–30 min run), indicating high purity (>95%). The inset UV–Vis spectrum displays a single λ_max at 254 nm, confirming spectral homogeneity and compound identity.

Analytical RP-HPLC evaluation of the purified fraction revealed a single, sharp, and symmetrical peak eluting at RT = 16.47 min, confirming successful isolation of thymoquinone with high chromatographic resolution. No additional peaks exceeding baseline noise were detected throughout the 5–30 min run, indicating the absence of co-eluting impurities as shown in Figure 3. The peak exhibited a narrow width and near-Gaussian profile, reflecting high column efficiency and minimal band broadening under the applied separation conditions.

Quantitative purity assessment using the peak area normalization method demonstrated that the principal peak accounted for >95% of the total integrated chromatographic area, confirming high chemical purity. Minor baseline fluctuations observed at early and late retention times were negligible and did not correspond to discrete impurity signals.

The inset UV–Vis spectrum of the eluted peak displayed a single absorption maximum (λ_max) at 254 nm, consistent with the characteristic π→π* electronic transitions of the quinone chromophore in thymoquinone. Spectral homogeneity across the chromatographic peak further corroborated compound identity and excluded the presence of spectrally distinct co-eluting contaminants. Following fraction collection and solvent removal under reduced pressure, 82 mg of highly purified thymoquinone was obtained, corresponding to a recovery yield of 68.3% relative to the injected TLC fraction. This purification procedure was repeated five independent times under identical preparative TLC and subsequent HPLC validation conditions, yielding approximately 82 mg per cycle and a cumulative total of ~410 mg (82 mg × 5).

### 2.4. Antibacterial and MICs of Purified Thymoquinone

**Figure 4:**
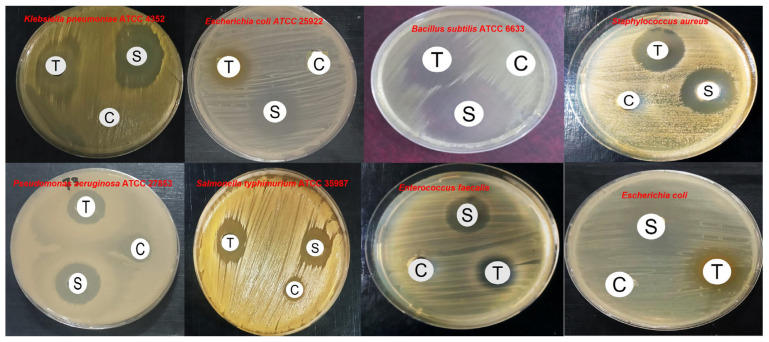
Representative disk diffusion assay demonstrating the antimicrobial activity of thymoquinone against reference and foodborne bacterial strains. Discs were designated as follows: C = negative control (DMSO), T = thymoquinone, and S = positive control (ciprofloxacin).

**Figure 5:**
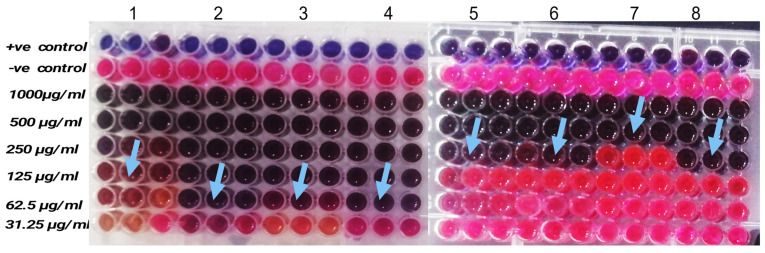
Representative MUCs of thymoquinone, against reference and foodborne bacterial strains. 1 = *Staphylococcus aureus*, 2 = *Bacillus subtilis* ATCC 6633, 3 = *Staphylococcus aureus* ATCC 29213, 4 = *Listeria monocytogenes*, 5 = *Escherichia coli* ATCC 25922, 6 = *Klebsiella pneumoniae* ATCC 4352, 7 = *Pseudomonas aeruginosa* ATCC 27853 and 8 = *Salmonella typhimurium* ATCC 35987.

The antibacterial activity of thymoquinone separated from *N. sativa* seeds was evaluated against a panel of Gram-positive and Gram-negative bacteria using inhibition zone diameter and MIC determinations (Table 3). Thymoquinone exhibited measurable antibacterial activity against all tested strains, although its efficacy was consistently lower than that of the reference antibiotic ciprofloxacin (5 µg/mL). Statistical analysis revealed that the inhibition zones produced by ciprofloxacin were significantly larger than those of thymoquinone across all bacterial species (*p* < 0.01–0.001), confirming the superior potency of the conventional antibiotic. Among Gram-positive bacteria, *Staphylococcus aureus* ATCC 29213 and *Bacillus subtilis* ATCC 6633 were the most susceptible to thymoquinone, with inhibition zones of 21.3 ± 1.0 mm and 20.5 ± 0.9 mm, respectively (Figure 4, and MIC values of 62.5 µg/mL. Similar susceptibility was observed for *Listeria monocytogenes* (22.6 ± 0.9 mm; MIC −62.5 µg/mL). In contrast, Gram-negative bacteria demonstrated comparatively reduced susceptibility. *Pseudomonas aeruginosa* ATCC 27853 showed the lowest sensitivity, with an inhibition zone of 13.4 ± 0.6 mm and a MIC of 500 µg/mL (Figure 5).

### 2.5. Antibiofilm Activity of Thymoquinone

**Figure 6:**
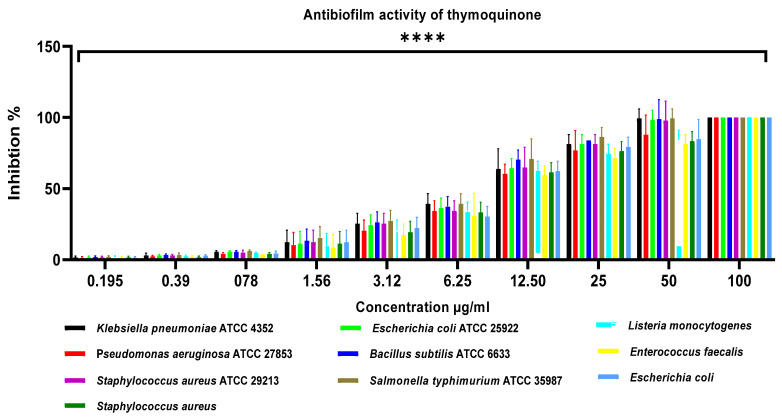
Antibiofilm activity of thymoquinone against different bacterial strains at increasing concentrations (0.195–100 µg/mL). Biofilm inhibition is expressed as percentage inhibition. Data are presented as mean ± SD, showing a significant concentration-dependent effect (**** *p* < 0.0001).

The antibiofilm activity of thymoquinone against a panel of Gram-positive and Gram-negative bacteria was quantitatively evaluated using the crystal violet assay, and the results are summarized in Figure 6. Thymoquinone exhibited a clear, concentration-dependent inhibition of biofilm formation across all tested bacterial strains, with concentrations ranging from 0.195 to 100 µg/mL.

At the lowest tested concentrations (≤0.78 µg/mL), thymoquinone induced only marginal biofilm inhibition (<10%) for most strains, indicating limited antibiofilm efficacy at sub-micromolar levels. However, a significant increase in biofilm inhibition was observed from 1.56 to 12.5 µg/mL, suggesting the onset of effective antibiofilm activity. At intermediate concentrations (6.25–12.5 µg/mL), biofilm inhibition ranged between approximately 35–70%, depending on the bacterial species, highlighting interspecies variability in susceptibility. At higher concentrations (25–50 µg/mL), thymoquinone achieved robust antibiofilm effects, with inhibition exceeding 80% for most organisms. Notably, near-complete inhibition (~100%) biofilm formation was observed at 100 µg/mL for all tested strains, including *Klebsiella pneumoniae* ATCC 4352, *Pseudomonas aeruginosa* ATCC 27853, *Staphylococcus aureus* ATCC 29213, *Listeria monocytogenes*, *Enterococcus faecalis*, and both reference and foodborne *Escherichia coli* strains. Statistical analysis revealed that the reductions in biofilm biomass at concentrations ≥6.25 µg/mL were highly significant (**** *p* < 0.0001) compared with untreated controls.

### 2.6. Antioxidant Activity of Thymoquinone Assessed by DPPH and ABTS Assays

The antioxidant capacity of thymoquinone was evaluated using DPPH and ABTS assays and compared with ascorbic acid over 7.81–1000 μg/mL (Table 4). Thymoquinone showed a concentration-dependent increase in radical scavenging, indicating its ability to neutralize free radicals. In the DPPH assay, scavenging activity ranged from 18.6 ± 1.4% at 7.81 μg/mL to 94.6 ± 4.6% at 1000 μg/mL, while ascorbic acid achieved 32.4 ± 1.6% to 98.9 ± 4.8% across the same range. Differences between thymoquinone and ascorbic acid were significant at all concentrations (*p* < 0.05–0.001). In the ABTS assay, overall scavenging was higher, with thymoquinone reaching 97.8 ± 4.4% and ascorbic acid 99.6 ± 4.7% at 1000 μg/mL, reflecting the ABTS method’s sensitivity to both hydrophilic and lipophilic antioxidants. IC_50_ values further illustrated the difference in potency: thymoquinone exhibited IC_50_s of 52.3 ± 2.1 μg/mL (DPPH) and 41.6 ± 1.9 μg/mL (ABTS), compared with 18.7 ± 1.4 μg/mL and 14.2 ± 1.2 μg/mL for ascorbic acid (*p* < 0.001).

### 2.7. Anti-Inflammatory Activity of Thymoquinone Assessed by HRBC Membrane Stabilization

The anti-inflammatory potential of thymoquinone was assessed using the HRBC membrane stabilization assay and compared with sodium diclofenac over 1–128 μg/mL (Table 5). Both compounds showed concentration-dependent membrane protection against hypotonicity-induced hemolysis. At 1 μg/mL, thymoquinone exhibited modest inhibition (12.4 ± 1.1%) compared with sodium diclofenac (18.6 ± 1.3%, *p* < 0.05), a trend maintained across all concentrations. At the highest concentration (128 μg/mL), thymoquinone achieved 85.6 ± 4.4% inhibition, versus 94.2 ± 4.7% for sodium diclofenac (*p* < 0.001). IC_50_ values further reflected these differences, with thymoquinone at ~14.8 μg/mL and sodium diclofenac at ~6.3 μg/mL, confirming the greater potency of the standard drug while highlighting the substantial intrinsic anti-inflammatory activity of thymoquinone.

### 2.8. Anti-Diabetic Activity of Thymoquinone

**Figure 7:**
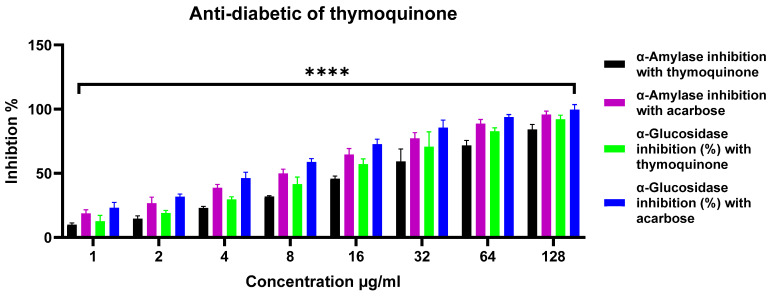
In vitro antidiabetic activity of thymoquinone. Percentage inhibition of α-amylase and α-glucosidase by thymoquinone at different concentrations (1–128 μg/mL) compared with the standard inhibitor acarbose. Data are presented as mean ± SD. Statistical significance between thymoquinone and acarbose-treated groups is indicated (**** *p* < 0.0001).

The anti-diabetic potential of thymoquinone was assessed via inhibition of α-amylase and α-glucosidase and compared with the standard drug acarbose (Figure 7). Both compounds showed concentration-dependent inhibition across 1–128 μg/mL, with acarbose exhibiting significantly higher potency at all concentrations (*p* < 0.05–0.001). Thymoquinone produced modest inhibition at low concentrations (α-amylase: 10–32%; α-glucosidase: 12–41%) and reached ~84% and ~92% inhibition of α-amylase and α-glucosidase, respectively, at 128 μg/mL, whereas acarbose achieved >95%. Notably, thymoquinone inhibited α-glucosidase more effectively than α-amylase, indicating selective enzyme inhibition and substantial anti-diabetic potential at moderate to high concentrations.

## 3. Discussion

Plant-derived natural products have long been utilized in the pharmaceutical, food, and cosmetic industries and are extensively documented across many cultures. Historically, ancient civilizations employed these resources both as dietary components and as therapeutic agents. In recent decades, research interest has grown substantially, focusing on the detailed characterization of their chemical composition and the exploration of their potential applications across diverse fields [1].

This study integrates comprehensive phytochemical profiling with biological evaluation of *N. sativa* seed extracts, with particular emphasis on thymoquinone as a key bioactive constituent. Extraction efficiency was strongly dependent on solvent polarity, with thymoquinone consistently isolated from the methanolic extract. UHPLC/QTOF-MS analysis enabled detailed chemical characterization of the extracts, followed by targeted purification using TLC and HPLC. Subsequent in vitro assays demonstrated the antibacterial, antibiofilm, antioxidant, anti-inflammatory, and enzyme-inhibitory activities of the purified thymoquinone.

Quantitative phytochemical analysis of *N. sativa* seeds was performed following sequential maceration with hexane, ethyl acetate, methanol, and ethanol, yielding crude extract recoveries of 5.5%, 6.2%, 8.4%, and 7.2% (*w*/*w*), respectively, and demonstrating pronounced solvent-dependent differences in metabolite recovery. These results are consistent with those reported by El-Dabeer et al. [22], who observed maximum yields of 7.5%, 4.4%, 3.8%, and 3.4% (*w*/*w*) for water, methanol, isopropanol, and hexane, respectively. Moreover, methanolic and ethanolic extracts contained significantly higher levels of phenols, flavonoids, alkaloids, and saponins than ethyl acetate and hexane extracts, corroborating previous findings that polar solvents more efficiently extract hydrophilic secondary metabolites, particularly phenolic compounds and flavonoids, from *N. sativa* seeds [29,30,31]. Phenolic compounds and flavonoids are known to possess strong antioxidant, anti-inflammatory, and antimicrobial properties, which may explain the enhanced biological activities often reported for methanolic and ethanolic extracts of *N. sativa* [30,32]. The high phenolic and flavonoid contents observed in the methanolic extract align with findings by Ahmad et al. [29], and Abbas et al. [30], who reported methanol as the most efficient solvent for recovering antioxidant compounds from *N. sativa*. Similarly, ethanol has been widely reported as an effective and biocompatible solvent for extracting bioactive phytochemicals, supporting its traditional and pharmaceutical relevance [30]. In contrast, hexane extract showed comparatively lower concentrations of polar phytochemicals, with saponins and tannins not detected. However, hexane yielded significantly higher levels of steroids and terpenoids, which are predominantly lipophilic compounds. This result is consistent with earlier studies demonstrating that non-polar solvents preferentially extract volatile oils, terpenes, and sterol fractions from *N. sativa* seeds [33,34]. Terpenoids and steroids have been associated with antimicrobial, anti-inflammatory, and immunomodulatory activities, suggesting that hexane extracts may still possess therapeutic relevance despite their lower phenolic content [34]. Ethyl acetate extract displayed intermediate phytochemical levels across most classes, reflecting its semi-polar nature. This pattern is in agreement with previous reports where ethyl acetate extracts showed moderate recovery of both polar and non-polar compounds [35]. However, some studies have reported higher flavonoid and phenolic contents in ethyl acetate extracts compared to methanol, highlighting that extraction efficiency may vary depending on seed origin, processing conditions, and analytical methods [36]. Such discrepancies may explain the quantitative differences observed between the present findings and certain earlier reports. Overall, the results of this study are consistent with existing literature, confirming methanol and ethanol as superior solvents for extracting phenolic-rich fractions from *N. sativa* seeds, while hexane is more suitable for isolating lipophilic constituents [34]. Variations in phytochemical yield across studies may be attributed to differences in geographical origin, climatic conditions, extraction protocols, and plant chemotypes [30,35].

UHPLC/QTOF-MS analysis identified thymoquinone as a major component of the methanolic extract, supporting its role as a characteristic marker compound of *N. sativa* seeds [37]. Numerous studies demonstrated the seed of *N. sativa* and its main active constituent, thymoquinone, to be medicinally very effective against various illnesses including different chronic: neurological and mental illness, cardiovascular disorders, cancer, diabetes, inflammatory conditions and infertility, as well as various infectious diseases due to bacterial, fungal, parasitic, and viral infections [6,31]. Additional terpenoids (e.g., *p*-cymene, carvacrol) and the alkaloid nigellidine were also detected, underscoring the chemical complexity of the extract. The presence of metabolites spanning several chemical classes highlights the suitability of high-resolution LC-MS for comprehensive profiling and is consistent with earlier studies describing the diverse phytochemical composition of *N. sativa* [38,39,40,41].

The isolation and purification of thymoquinone from *N. sativa* methanolic extract in this study demonstrate both the efficiency of the extraction process and the robustness of the chromatographic strategies employed. Initial isolation using TLC yielded 270 mg/g (27.0%) of thymoquinone, which is consistent with previously reported yields from methanolic extracts (28.62%) [21], but higher than other reports, which typically range from 1–10% depending on extraction conditions and plant source [32,33,34]. This yield highlights methanol’s effectiveness as a polar solvent for selectively extracting thymoquinone, in agreement with earlier studies emphasizing solvent polarity as a critical factor in maximizing quinone recovery. Overall, these findings underscore the critical influence of extraction conditions and solvent choice on the recovery of bioactive compounds [33,34]. Subsequent purification using RP-HPLC enhanced the purity of thymoquinone to >95%, yielding 82 mg with a 68.3% recovery from the TLC-enriched fraction. Compared with other reported purification strategies, including column chromatography and preparative HPLC alone, the combined TLC–HPLC approach offers several advantages: it allows preliminary enrichment of the target compound, reduces co-elution of minor constituents, minimizes solvent consumption, and ensures high reproducibility and scalability. The relatively high recovery rate also indicates that this stepwise method effectively balances purity and yield, providing sufficient quantities of thymoquinone for subsequent in vitro assays while minimizing compound loss. These results are consistent with the well-established effect of solvent polarity on extraction efficiency, where polar solvents favor hydrophilic secondary metabolites and non-polar solvents enrich lipophilic constituents [29,30,31,32,33,34]. TLC separation enabled effective resolution and recovery of thymoquinone, yielding a distinct band with an Rf value consistent with published data [29]. The appearance of additional minor bands reflects the co-extraction of structurally related compounds under methanolic conditions [31,34]. The concordance between TLC and UHPLC/QTOF-MS results validates the chromatographic conditions and confirms the suitability of TLC and HPLC as sequential purification steps prior to advanced chemical and biological analyses [32,33].

In the present study, purified thymoquinone exhibited antibacterial activity against both Gram-positive and Gram-negative bacteria, with Gram-positive strains showing markedly higher susceptibility. This differential activity reflects fundamental differences in bacterial cell envelope architecture, as the absence of an outer membrane in Gram-positive bacteria facilitates the penetration of hydrophobic compounds such as thymoquinone [42,43]. In contrast, the reduced sensitivity of Gram-negative bacteria, particularly *Pseudomonas aeruginosa*, can be attributed to intrinsic resistance mechanisms, including limited membrane permeability and active efflux systems [44,45,46]. Previous studies have reported that thymoquinone isolated from *N. sativa* seeds exerts broad-spectrum antibacterial effects against a wide range of Gram-positive and Gram-negative bacteria, including *Bacillus*, *Listeria*, *Enterococcus*, *Micrococcus*, *Staphylococcus*, *Pseudomonas*, *Escherichia*, *Salmonella*, and *Vibrio parahaemolyticus*, as well as strong inhibition of bacterial biofilm formation [6]. Similarly, methanolic extracts of *N. sativa* seeds have demonstrated greater antibacterial activity against Gram-positive bacteria, such as *Streptococcus pyogenes*, compared with Gram-negative species, including *P. aeruginosa*, *Klebsiella pneumoniae*, and *Proteus vulgaris* [42,43]. Additionally, *N. sativa* oils at various concentrations have shown substantial inhibitory effects against multiple methicillin-resistant *Staphylococcus aureus* (MRSA) isolates. Thymoquinone itself exhibits potent bactericidal activity against Gram-positive cocci, with minimum inhibitory concentrations (MICs) ranging from 8 to 32 μg/mL, and effectively suppresses biofilm formation, with minimum biofilm inhibitory concentrations of 22 μg/mL for *S. aureus* and 60 μg/mL for *S. epidermidis* [6].

Thymoquinone also inhibited biofilm formation across all tested strains, with MBIC_50_ values lower than the corresponding MICs, indicating activity at sub-inhibitory concentrations. Gram-positive bacteria showed slightly greater sensitivity than Gram-negative species, consistent with differences in biofilm architecture and matrix composition. These observations are in line with earlier studies reporting that thymoquinone interferes with early biofilm development and biofilm-associated regulatory processes [11,47,48]. Furthermore, *N. sativa* seed extracts and thymoquinone were found to suppress *S. aureus* biofilm formation, a key contributor to the spread of community-acquired *S. aureus* infections and the persistence of chronic infections, as biofilms enhance bacterial resistance to host defense mechanisms [11].

Oxidative stress, characterized by excessive production of free radicals, is a major contributor to the pathogenesis of numerous progressive disorders, including neurodegenerative diseases, cancer, aging, and endocrine dysfunctions. Accordingly, there is growing interest in medicinal plants as sources of natural antioxidants with therapeutic potential [1]. In the present study, thymoquinone exhibited measurable radical scavenging activity in both DPPH and ABTS assays, although its potency was lower than that of ascorbic acid. The lower IC_50_ values observed in the ABTS assay compared with the DPPH assay are consistent with known methodological differences between these systems, particularly regarding radical solubility and accessibility to lipophilic antioxidants [41]. While such chemical assays are valuable for comparative evaluation, they may not fully reflect antioxidant behavior under biological conditions [49]. Supporting these findings, methanolic extracts and essential oil fractions derived from *N. sativa* seeds have been reported to significantly enhance plasma total antioxidant capacity in rats an atherogenic diet, achieving up to an 88% improvement in free radical scavenging activity. Furthermore, administration of *N. sativa* oil and thymoquinone has been shown to attenuate cisplatin-induced disturbances in carbohydrate metabolism and restore both enzymatic and nonenzymatic antioxidant defense systems in the gastric mucosa [6].

Inflammation is a central pathological process underlying numerous disorders, including cystic fibrosis, rheumatoid arthritis, osteoarthritis, asthma, allergic diseases, and cancer, many of which are associated with acute or chronic pain. Although conventional anti-inflammatory therapies are effective, their prolonged use is frequently limited by serious adverse effects, such as gastrointestinal ulceration, bone marrow suppression, and fluid and electrolyte imbalance. Consequently, medicinal plants, including *N. sativa*, represent promising sources of anti-inflammatory agents with improved safety profiles and reduced side effects [6]. In the present study, thymoquinone exhibited concentration-dependent anti-inflammatory activity in the human red blood cell (HRBC) membrane stabilization assay, albeit with lower efficacy than diclofenac sodium. Membrane stabilization is widely accepted as an indicator of anti-inflammatory potential due to the structural and functional similarities between erythrocyte and lysosomal membranes involved in inflammatory responses [50,51]. The observed activity is consistent with previous reports describing the anti-inflammatory effects of thymoquinone in experimental models [52,53,54,55,56]. Notably, natural anti-inflammatory compounds typically display IC_50_ values in the range of 5–50 μg/mL [49,50], and the IC_50_ value of thymoquinone in the present study (~14.8 μg/mL) falls well within this range, in agreement with earlier findings for plant-derived quinones and phenolic compounds. In support of these results, alcoholic extracts of *N. sativa* have previously demonstrated significant analgesic activity in murine models, in some cases comparable to or exceeding that of diclofenac sodium. The anti-inflammatory effects of thymoquinone are thought to be mediated, at least in part, through inhibition of arachidonic acid metabolism, specifically by suppressing the production of pro-inflammatory mediators such as thromboxane B_2_ and leukotrienes via dual inhibition of cyclooxygenase and lipoxygenase pathways [6].

In this study, thymoquinone inhibited both α-amylase and α-glucosidase, with greater inhibitory activity toward α-glucosidase. Such selectivity is considered relevant, as preferential α-glucosidase inhibition may reduce gastrointestinal side effects associated with excessive α-amylase inhibition [28]. Although IC_50_ values were higher than those of acarbose, the dose-dependent inhibition observed supports a reproducible, moderate enzyme inhibitory effect. Reported interactions between quinone structures and enzyme active or regulatory sites may contribute to this activity [57,58]. In the context of its combined antioxidant and anti-inflammatory properties, these findings support further investigation of thymoquinone as a multifunctional natural compound, while acknowledging the limitations inherent to in vitro enzyme assays [49,59]. Administration of *N. sativa* seeds to streptozotocin (STZ)-induced diabetic rats for one month resulted in a significant reduction in fasting plasma glucose levels, serum malondialdehyde (MDA), interleukin-6, and immunoglobulins (IgA, IgG, and IgM), accompanied by a marked enhancement of endogenous antioxidant defenses, including superoxide dismutase (SOD), glutathione-S-transferase, and catalase. Histopathological examination of the pancreas in *N. sativa*-treated animals further demonstrated notable improvements in β-cell degeneration, inflammation, and vascular congestion compared with diabetic controls [6,28]. Similarly, combined administration of *N. sativa* and *Cinnamomum cassia* extracts to STZ-induced diabetic rats significantly stabilized serum glucose levels, lipid profiles, and renal function parameters, with more pronounced effects observed when the extracts were co-administered with metformin. This treatment regimen also led to substantial reversal of pancreatic histopathological damage [6,59]. In clinical settings, three-month supplementation with *N. sativa* (2 g/day) as an adjunct to oral antidiabetic therapy in patients with type 2 diabetes mellitus has been reported to significantly reduce glycated hemoglobin, and thiobarbituric acid reactive substances, while significantly enhancing total antioxidant capacity, SOD activity, and glutathione levels [60].

Collectively, these findings clarify associations between extract composition and observed bioactivity under controlled in vitro conditions. This integrative approach underscores the pharmacological significance of *N. sativa* and provides a foundation for future studies aimed at developing complementary or adjuvant treatments.

## 4. Materials and Methods

### 4.1. Collection Preparation of N. sativa Seed Extracts

*Nigella sativa* seeds were obtained from local market in April 2025 from the Hail region in Saudi Arabia. The collected seeds were thoroughly rinsed with distilled water to remove surface contaminants and debris. They were subsequently air-dried at ambient laboratory temperature (28 ± 2 °C) under shaded conditions to prevent photodegradation of thermolabile and photosensitive compounds. Drying was carried out for a period of one week, during which the seeds were turned periodically to ensure uniform moisture loss. Once fully dried, the seeds were mechanically ground into a fine powder using a sterilized electric grinder. The powdered material was immediately transferred into sterile, airtight containers and stored in a cool, dry environment until extraction.

Solvent extraction was performed using a weight-to-volume ratio of 1:5 (*w*/*v*). Briefly, 100 g of powdered seed material was macerated separately in 500 mL of each of the following analytical-grade solvents: hexane (non-polar), ethyl acetate (semi-polar), methanol (polar), and ethanol (polar). These solvents were selected based on their polarity indices to maximize the extraction efficiency of chemically diverse phytoconstituents, including lipophilic and hydrophilic compounds. Maceration was conducted in amber-colored glass bottles at room temperature for 72 h with intermittent manual agitation to enhance solvent penetration and mass transfer. The mixtures were filtered every 24 h using Whatman No. 1 filter paper, and the resulting filtrations were pooled. Solvent removal was carried out under reduced pressure using a rotary evaporator (Heidolph VV200, Schwabach, Germany) set at 45 °C to prevent thermal degradation of bioactive compounds [61]. The concentrated crude extracts were weighed to determine extraction yield and stored at 4 °C in sterile containers until further use.

### 4.2. Quantitative Determination Phytochemical

The crude extracts obtained using hexane (HE), ethyl acetate (EAE), methanol (ME), and ethanol (EE) were subjected to quantitative determination of major phytochemical classes, including phenols, flavonoids, alkaloids, saponins, tannins, steroids, and terpenoids, using established spectrophotometric and gravimetric methods [62,63,64]. All absorbance measurements were performed using a UV–visible spectrophotometer (UV-1800, Shimadzu Corporation, Kyoto, Japan). All assays were conducted in triplicate, and results were expressed as mean ± standard deviation.

#### 4.2.1. Determination of Total Saponin Content

Total saponin content was quantified using the gravimetric method described by Nagori et al. [62]. Briefly, a measured volume (5 mL) of each extract was subjected to liquid–liquid partitioning with n-butanol. The combined n-butanol fractions were subsequently washed with 5% (*w*/*v*) sodium chloride solution to remove impurities, then evaporated to dryness under reduced pressure. The resulting saponin-rich residue was dried to constant weight and accurately weighed. Total saponin content was calculated gravimetrically according to the following equation:

$$\mathrm{Saponins}\;(\mathrm{mg}/\mathrm{mL})=\frac{\mathrm{Weight}\;\mathrm{of}\;\mathrm{dried}\;\mathrm{saponin}\;\mathrm{residue}\;(\mathrm{mg})}{V\;(\mathrm{mL})}$$

where *V* represents the volume of extract used for analysis. Results were expressed as mg of saponins per mL of extract. All measurements were performed in triplicate, and data were reported as mean ± standard deviation (SD).

#### 4.2.2. Determination of Total Alkaloid Content

Total alkaloid content was quantified using the gravimetric method described by Nagori et al. [62]. Briefly, a measured volume (5 mL) of each extract was acidified with 2% (*v*/*v*) acetic acid in ethanol and allowed to stand for 4 h to ensure complete alkaloid extraction. The mixture was then filtered, and the filtrate was concentrated under reduced pressure. Concentrated ammonium hydroxide was added dropwise to the concentrated solution to induce alkaloid precipitation. The resulting precipitate was collected, washed with diluted ammonium hydroxide to remove residual impurities, dried to constant weight, and accurately weighed. Total alkaloid content was calculated gravimetrically using the following equation:

$$\mathrm{Alkaloids}\;(\mathrm{mg}/\mathrm{mL})=\frac{\left(W_{2}-W_{1}\right)}{V}$$

where $W_{2}$ = weight of container plus dried alkaloid residue (mg), $W_{1}$ = weight of empty container (mg), V = volume of extract used for analysis (mL). Results were expressed as mg of alkaloids per mL of extract. All determinations were performed in triplicate, and data were reported as mean ± standard deviation (SD).

#### 4.2.3. Determination of Total Tannin Content

Total tannin content was determined spectrophotometrically using the Folin–Denis method as described by Nagori et al. [62]. Briefly, an aliquot of the extract (5 mL) was mixed with Folin–Denis reagent, followed by the addition of sodium carbonate solution to develop color. The reaction mixture was incubated under controlled conditions to allow complete chromogen formation. A reagent blank was prepared simultaneously by replacing the sample or standard with 0.5 mL of distilled water. Absorbance was measured at 760 nm using a UV–Vis spectrophotometer. Quantification was performed using a calibration curve constructed with tannic acid standard solutions of known concentrations. Results were expressed as milligrams of tannic acid equivalents per mL of extract (mg TAE/mL). Total tannin content was calculated according to the following equation:

$$\mathrm{Tannins}\;(\mathrm{mg}\;\mathrm{TAE}/\mathrm{mL})=\frac{C\times V}{V_{s}}$$

where C = concentration of tannic acid equivalent obtained from the calibration curve (mg/mL), V = total volume of the reaction mixture (mL), $V_{s}$ = volume of extract used in the assay (mL). All measurements were performed in triplicate, and results were reported as mean ± standard deviation (SD).

#### 4.2.4. Determination of Total Flavonoids Content (TFC)

Total flavonoid content was quantified using the aluminum chloride colorimetric method as described by El-Sherbiny et al. [1]. Briefly, 0.5 mL of each extract (*V_s_*) was mixed with 0.1 mL of 10% (*w*/*v*) AlCl_3_, 0.1 mL of 1 M potassium acetate, and 2.8 mL of distilled water to obtain a final reaction volume (*V*). The mixture was incubated at room temperature for 30 min to allow formation of the flavonoid–aluminum complex. A reagent blank was prepared under identical conditions by substituting the extract or standard with 0.5 mL of distilled water. Absorbance was measured at 415 nm using a UV–Vis spectrophotometer. Quantification was performed using a calibration curve constructed with quercetin standard solutions (20–80 μg/mL). The flavonoid concentration (*C*) of each sample was interpolated from the linear regression equation of the standard curve and expressed as milligrams of quercetin equivalents per mL of extract (mg QE/mL).

Total flavonoid content was calculated using the following equation:

$$\mathrm{TFC}\;(\mathrm{mg}\;\mathrm{QE}/\mathrm{mL})=\frac{C\times V}{V_{s}}$$

where C = quercetin-equivalent concentration derived from the calibration curve (mg/mL), V = total reaction volume (mL), $V_{s}$ = volume of extract used in the assay (mL).

All measurements were performed in triplicate, and results were expressed as mean ± standard deviation (SD).

#### 4.2.5. Determination of Total Phenolic Content (TPC)

Total phenolic content was determined using the Folin–Ciocalteu colorimetric method as described by Shawky et al. [64]. Briefly, 0.5 mL of each extract (*V_s_*) was mixed with 2.5 mL of 10% (*v*/*v*) Folin–Ciocalteu reagent and allowed to react for 5 min. Subsequently, 2.0 mL of 7.5% (*w*/*v*) sodium carbonate solution was added. The reaction mixture was incubated at room temperature for 30 min to ensure complete color development. A reagent blank was prepared under identical conditions by replacing the extract or standard with 0.5 mL of distilled water. Absorbance was measured at 765 nm using a UV–Vis spectrophotometer. Quantification was performed using a calibration curve constructed with gallic acid standard solutions (10–100 µg/mL). The calibration equation was expressed as:

y=ax+b

where *y* represents absorbance and *x* represents gallic acid concentration (µg/mL). The phenolic concentration of each sample (*C*) was calculated by interpolating its absorbance value from the regression equation and converting to mg/mL. Total phenolic content was calculated according to the following equation:

$$\mathrm{TPC}\;(\mathrm{mg}\;\mathrm{GAE}/\mathrm{mL})=\frac{C\times V}{V_{s}}$$

where C = concentration obtained from the calibration curve (mg/mL), V = total reaction volume (mL), $V_{s}$ = volume of extract used in the assay (mL). Results were expressed as milligrams of gallic acid equivalents per mL of extract (mg GAE/mL). All measurements were performed in triplicate and reported as mean ± standard deviation (SD).

#### 4.2.6. Determination of Total Steroids

Total steroid content was quantified spectrophotometrically according to the method described by Nagori et al. [62]. Briefly, 0.5 mL of each extract (*V_s_*) was mixed with 2.0 mL of acetic anhydride, followed by the careful addition of 0.5 mL of concentrated sulfuric acid to initiate color development. The reaction mixture was incubated at room temperature for 15 min to allow complete formation of the chromogenic complex. A reagent blank was prepared under identical conditions by replacing the extract or standard with 0.5 mL of distilled water. Absorbance was measured at 620 nm using a UV–Vis spectrophotometer.

Quantification was performed using a calibration curve constructed with cholesterol standard solutions (20–80 µg/mL). The calibration curve was generated by plotting absorbance versus cholesterol concentration, and the regression equation was used to determine the equivalent steroid concentration (*C*) of each sample. Total steroid content was calculated according to the following equation:

$$\mathrm{Steroids}\;(\mathrm{mg}\;\mathrm{CE}/\mathrm{mL})=\frac{C\times V}{V_{s}}$$

where C = cholesterol-equivalent concentration obtained from the calibration curve (mg/mL), V = total reaction volume (mL), $V_{s}$ = volume of extract used in the assay (mL). Results were expressed as milligrams of cholesterol equivalents per mL of extract (mg CE/mL). All analyses were performed in triplicate and reported as mean ± standard deviation (SD).

#### 4.2.7. Determination of Total Terpenoids

Total terpenoid content was quantified using the gravimetric method described by Nagori et al. [62], based on solvent extraction followed by residue weighing. Briefly, a measured volume (5 mL) of each extract was subjected to appropriate solvent extraction to isolate the terpenoid fraction. The solvent was then evaporated to dryness under reduced pressure, and the resulting residue was dried to constant weight to ensure complete solvent removal. The dried terpenoid residue (*W_t_*) was accurately weighed using an analytical balance. Total terpenoid content was calculated gravimetrically according to the following equation:

$$\mathrm{Terpenoids}\;(\mathrm{mg}/\mathrm{mL})=\frac{W_{t}}{V}$$

where $W_{t}$ = weight of dried terpenoid residue (mg), V = volume of extract used for analysis (mL). Results were expressed as milligrams of terpenoids per mL of extract. All determinations were performed in triplicate and reported as mean ± standard deviation (SD).

### 4.3. Chemical Analysis of the N. sativa Methanolic Extract Using UHPLC/QTOF-MS

#### 4.3.1. Chemicals and Reagents

LC-MS-grade acetonitrile and gradient grade solvents, including isopropanol, methanol, dichloromethane, and ethyl acetate, were obtained from Thermo Fisher Scientific (Waltham, MA, USA). Formic acid (98%), ammonium hydroxide, ammonium formate, and ammonium acetate were purchased from Sigma-Aldrich (St. Louis, MO, USA). All reagents and solvents were of analytical or LC–MS grade and were used without further purification.

#### 4.3.2. Sample Preparation

The dried *N. sativa* methanolic extract (50 mg) was reconstituted in 1000 µL of reconstitution solvent composed of water:methanol:acetonitrile (2:1:1, *v*/*v*). The mixture was vortexed for 2 min, followed by ultrasonication at 30 kHz for 10 min to ensure complete dissolution. An aliquot of 20 µL from the resulting stock solution (50 mg/1000 µL) was further diluted with 980 µL of the same reconstitution solvent, centrifuged at 10,000 rpm for 5 min, and filtered through a 0.22 µm PTFE syringe filter prior to UHPLC/QTOF-MS analysis. The clear supernatant was transferred into LC-MS vials. The injection volume was set at 10 µL, corresponding to a final sample concentration of 1 µg/µL. Blank and quality control (QC) samples were prepared to ensure analytical reliability and data quality. The blank consisted solely of the reconstitution solvent (water:methanol:acetonitrile, 2:1:1, *v*/*v*) and was used to monitor background signals and potential carryover. QC samples were prepared by dissolving authentic thymoquinone standard in the same reconstitution solvent at defined concentrations within the analytical range. These QC samples were analyzed intermittently throughout the run to verify system performance, retention time stability, and signal reproducibility.

#### 4.3.3. Instrumentation and UHPLC–QTOF-MS Conditions

Chromatographic separation of small molecules was carried out using an Axion AC UHPLC system (Kyoto, Japan) equipped with an autosampler, an in-line filter disk pre-column (0.5 µm × 3.0 mm, Phenomenex, Torrance, CA, USA), and an XBridge C18 column (3.5 µm, 2.1 × 50 mm; Waters Corporation, Milford, MA, USA). The column temperature was maintained at 40 °C, and the flow rate was set at 300 µL/min. UHPLC separation was carried out using a binary mobile phase system. For analysis in the positive electrospray ionization (ESI^+^) mode, mobile phase (A) consisted of 1% methanol in 5 mM ammonium formate buffer, adjusted to pH 3.0 with formic acid, while mobile phase (B) was 100% acetonitrile. For the negative electrospray ionization (ESI^−^) mode, mobile phase (C) consisted of 1% methanol in 5 mM ammonium formate, adjusted to pH 8.0 with ammonium hydroxide.

In positive ion mode, chromatographic separation was performed using an A:B gradient elution program as follows: the initial composition was 10:90 (A:B, *v*/*v*; 90% B), which was linearly decreased to 10% B over 20 min. The mobile phase was then maintained at 10% B from 20 to 21 min, increased to 90% B from 21 to 25 min, decreased back to 10% B from 25 to 28 min, and finally returned to 90% B to allow column re-equilibration. In negative ion mode, separation was conducted under isocratic conditions using mobile phase C. Isocratic elution was selected to ensure stable retention and consistent deprotonation of acidic compounds, providing sharp peaks, reproducible retention times, and reliable quantitation while minimizing ionization variability associated with gradient elution.

Mass spectrometric detection was carried out using a TripleTOF™ 5600+ system equipped with a DuoSpray™ electrospray ionization (ESI) source (AB SCIEX, Concord, ON, Canada). Ion spray voltage and declustering potential were set at +4500 V/80 V in positive mode and −4500 V/−80 V in negative mode. The source temperature was maintained at 600 °C. Nitrogen was used as curtain gas (25 psi) and as nebulizer and auxiliary gases (Gas 1 and Gas 2, 40 psi each). Collision energy was fixed at ±35 V (mode-dependent) with a collision energy spread of 20 V and a mass accuracy tolerance of 10 ppm. Data were acquired in information-dependent acquisition (IDA) mode, enabling high-resolution full-scan MS (*m*/*z* 50–1100; 50 ms accumulation time) followed by automated MS/MS fragmentation of the most intense precursor ions. Instrument control and data acquisition were performed using Analyst TF v1.7 [63,65].

#### 4.3.4. Data Processing and Compound Annotation

Non-targeted metabolomic data were processed using MS-DIAL v3.70. Compound annotation was conducted against the ReSpect positive (2737 entries) or ReSpect negative (1573 entries) spectral libraries, depending on ionization polarity. Peak detection employed MS1 and MS2 tolerances of 0.01 Da and 0.05 Da, respectively, with a minimum peak height of 100 amplitude units, 0.05 Da mass slice width, two-scan smoothing, and a minimum peak width of six scans. For identification, MS1 and MS2 tolerances were set to 0.2 Da, with retention time alignment tolerance of 0.05 min and MS1 tolerance of 0.25 Da. Putative annotations were further verified using PeakView 2.2 with the MasterView 1.1 package (AB SCIEX). Feature validation was based on total ion chromatogram (TIC) inspection, applying acceptance criteria of signal-to-noise ratio >5 and significant sample-to-blank intensity discrimination, ensuring confident structural assignment supported by MS/MS fragmentation patterns [66].

### 4.4. Isolation and Purification of Thymoquinone from N. sativa Methanolic Extract

#### 4.4.1. Thin Layer Chromatography (TLC)

Thymoquinone in the *N. sativa* methanolic extract was analyzed and isolated using preparative TLC on pre-coated silica gel 60G plates (20 × 20 cm; Merck, Darmstadt, Germany). Chromatographic separation was performed using *n*-hexane: ethyl acetate (8:2, *v*/*v*) as the mobile phase in a saturated development chamber.

Aliquots of the methanolic extract and an authentic thymoquinone reference standard (Sigma-Aldrich) were applied as discrete bands, and the plates were developed until the solvent front approached the upper margin. Thymoquinone was identified by direct visual comparison with the reference standard and by matching retention factor (Rf) values. The thymoquinone band in the extract consistently exhibited an Rf value of 0.56, identical to that of the standard, thereby confirming its identity [67].

The corresponding silica gel band was carefully excised and eluted with methanol. Preparative TLC was repeated multiple times to isolate sufficient quantities of thymoquinone.

#### 4.4.2. Reverse-Phase High-Performance Liquid Chromatography (RP-HPLC)

Thymoquinone, preliminarily isolated by preparative TLC, was subjected to further purification and scale-up using semi-preparative RP-HPLC (Shimadzu SPD-10A, Kyoto, Japan). Chromatographic separation was achieved on a C18 semi-preparative column (250 mm × 10 mm i.d., 5 µm particle size) under optimized gradient elution conditions to ensure high resolution and reproducibility.

The mobile phase was prepared using solvent A as an aqueous solution of acetic acid at 1% *v*/*v* (i.e., 1 mL of glacial acetic acid per 100 mL of water) and solvent B (acetonitrile, HPLC grade). The acidic aqueous phase was employed to suppress secondary interactions and residual ionization effects, thereby enhancing peak symmetry, minimizing tailing, and improving retention stability. A linear gradient was applied as follows: 0–15 min, 15% B; 15–30 min, 45% B; and 30–45 min, 100% B, allowing efficient elution according to increasing hydrophobicity. After each run, the column was re-equilibrated to initial conditions to ensure retention time consistency across injections.

The flow rate was maintained at 4.0 mL/min, appropriate for the 10 mm internal diameter semi-preparative column, providing optimal separation efficiency while maintaining acceptable backpressure. Injection volumes ranged from 200 to 500 µL, depending on sample concentration and column loading capacity, to prevent overloading and band broadening. The column temperature was maintained at a constant 28 ± 2 °C using a thermostatted column oven to ensure reproducible retention times and chromatographic stability. Detection was performed at 254 nm using a UV detector, corresponding to the characteristic absorption maximum of thymoquinone.

The TLC-purified fraction was dissolved in HPLC-grade methanol, filtered through a 0.45 µm PTFE membrane filter, and injected into the system. Under the optimized conditions, thymoquinone eluted as a sharp, symmetrical, and well-resolved peak with a retention time of 16.47 min. The retention behavior and UV absorption spectrum were consistent with those of an authenticated thymoquinone reference standard, which was subsequently employed in all downstream biological assays.

Fractions corresponding to the target thymoquinone peak were collected using an automated fraction collector, with collection windows optimized according to injection volume and chromatographic performance. Multiple sequential injections were performed under identical operating conditions to maximize recovery while maintaining column integrity and separation efficiency. Collected fractions were pooled, concentrated under reduced pressure at controlled low temperature to prevent thermal degradation, gently evaporated under a nitrogen stream, and stored in amber vials at 4 °C to minimize oxidative and photochemical degradation [1,68]. Chemical purity was initially determined by peak area normalization using the equation:

Purity (%) = (Area of thymoquinone peak/Total integrated peak area) × 100

To further ensure chromatographic homogeneity, peak purity was assessed by photodiode array (PDA/DAD) spectral overlay analysis across the upslope, apex, and downslope of the peak. The absence of spectral deviation confirmed the lack of co-eluting impurities. The principal chromatographic peak represented >95% of the total integrated area, demonstrating high chemical purity suitable for structural confirmation and subsequent biological evaluation. Reproducible semi-preparative RP-HPLC runs were conducted to obtain sufficient quantities of analytically pure thymoquinone for downstream experimental studies.

### 4.5. Antibacterial Activity of Thymoquinone

#### 4.5.1. Disc Diffusion Assay

The antibacterial activity of the purified thymoquinone was evaluated against a panel of reference and foodborne bacterial strains, including *Salmonella typhimurium* ATCC 35987, *Bacillus subtilis* ATCC 6633, *Staphylococcus aureus* ATCC 29213, *Pseudomonas aeruginosa* ATCC 27853, *Klebsiella pneumoniae* ATCC 4352, and *Escherichia coli* ATCC 25922. In addition, four foodborne bacterial isolates (*Staphylococcus aureus*, *Escherichia coli*, *Enterococcus faecalis* and *Listeria monocytogenes*) obtained from the Faculty of Science, Al-Azhar University, were included in the analysis. Bacterial strains were initially cultured in Mueller-Hinton broth (MHB) and incubated at 37 °C for 24 h. The bacterial suspensions were then adjusted to a turbidity equivalent to 0.5 McFarland standard. Aliquots of 100 µL of each standardized suspension were evenly spread onto Mueller-Hinton agar (MHA) plates using sterile swabs. Sterile paper discs were impregnated with 100 µL of purified thymoquinone (1 mg/mL) dissolved in dimethyl sulfoxide (DMSO) and aseptically placed onto the surface of the inoculated agar plates. Ciprofloxacin (5 µg/mL) served as the positive control, while discs containing the extraction solvent alone were used as negative controls. The plates were incubated at 37 °C for 24 h under aerobic conditions. Following incubation, antibacterial activity was assessed by measuring the diameter of the inhibition zones surrounding each disc, expressed in millimeters. All assays were performed in triplicate, and the results are reported as mean ± standard deviation (SD) [1,69].

#### 4.5.2. Determination of Minimum Inhibitory Concentration

The MICs of ciprofloxacin hydrochloride monohydrate) (HiMedia Laboratories Pvt. Ltd., Mumbai, India).and the purified thymoquinone were determined using the broth microdilution method in sterile 96-well microplates, in accordance with the Clinical and Laboratory Standards Institute guidelines (CLSI, M7-A7). Briefly, Müller-Hinton broth (MHB) was inoculated with standardized bacterial suspensions of *Salmonella typhimurium* ATCC 35987, *Bacillus subtilis* ATCC 6633, *Staphylococcus aureus* ATCC 29213, *Pseudomonas aeruginosa* ATCC 27853, *Klebsiella pneumoniae* ATCC 4352, and *Escherichia coli* ATCC 25922. In addition, four foodborne bacterial isolates (*Staphylococcus aureus*, *Escherichia coli*, *Enterococcus faecalis* and *Listeria monocytogenes*) were included to achieve a final concentration of 1 × 10^6^ CFU/mL. Aliquots of 200 μL of the inoculated medium were dispensed into each well of the microplate. Ciprofloxacin and the purified thymoquinone were evaluated using two-fold serial dilutions. Ciprofloxacin was tested at concentrations ranging from 0.195 to 50 μg/mL, while the thymoquinone was assessed over a concentration range of 0.195 to 1000 μg/mL. Negative control wells containing MHB supplemented with the respective test compounds but lacking bacterial inoculation were included to correct for background absorbance. Following incubation at 37 °C for 18 h, bacterial growth was quantified by measuring absorbance at 630 nm using a microplate reader. The MIC was defined as the lowest concentration of ciprofloxacin or purified thymoquinone that resulted in complete inhibition of visible bacterial growth [62].

#### 4.5.3. Antibiofilm Properties of Purified Thymoquinone

The antibiofilm activity of thymoquinone was evaluated using the crystal violet microtiter plate assay, as previously described by Palanisamy et al. [11], with minor modifications. Bacterial cultures mentioned above were grown in Luria–Bertani (LB) broth and adjusted to a standardized inoculum of approximately 1 × 10^8^ CFU/mL. Aliquots of 100 µL of the standardized bacterial suspension were dispensed into sterile 96-well flat-bottom microplates containing thymoquinone at concentrations ranging from 1.95 to 100 µg/mL, prepared by two-fold serial dilution in LB broth. The plates were incubated statically at 37 °C for 24 h to allow biofilm formation. Following incubation, planktonic cells were gently removed, and the wells were washed three times with sterile phosphate-buffered saline (PBS) to eliminate non-adherent bacteria. The remaining biofilms were fixed and stained by adding 0.1% (*w*/*v*) crystal violet solution to each well and incubating at room temperature for 15 min. Excess stain was carefully discarded, and the wells were rinsed thoroughly with sterile PBS to remove unbound dye. The bound crystal violet was subsequently solubilized by adding 100 µL of absolute ethanol to each well. Biofilm biomass was quantified by measuring absorbance at 595 nm using a microplate reader (VMax, Molecular Devices, Sunnyvale, CA, USA). Wells containing bacterial cultures without thymoquinone served as positive control, while wells containing only sterile medium were used as blanks. The percentage inhibition of biofilm formation was calculated using the following equation:

$$\mathrm{Biofilm}\;\mathrm{inhibition}\;(\%)=\left(\frac{A_{\mathrm{control}}-A_{\mathrm{sample}}}{A_{\mathrm{control}}}\right)\times 100.$$

All experiments were performed in triplicate, and results were expressed as mean ± standard deviation (SD).

### 4.6. Antioxidant Activity of Purified Thymoquinone

#### 4.6.1. DPPH Radical Scavenging Assay

The free radical scavenging capacity of the purified thymoquinone was evaluated using the 2,2-diphenyl-1-picrylhydrazyl (DPPH) assay, following the method described by El-Sherbiny et al. [70]. Serial dilutions of thymoquinone were prepared at concentrations ranging from 7.81 to 1000 μg/mL. One hundred microliters of each concentration were mixed with 100 μL of a freshly prepared 0.1 mmol/L DPPH solution in methanol. Ascorbic acid at the same concentrations was used as the reference antioxidant, and methanol served as the blank. The reaction mixtures were incubated in the dark at 27 °C for 20 min, after which the decrease in absorbance was measured at 517 nm using a UV-Vis spectrophotometer. The antioxidant activity was expressed as the percentage of DPPH radical scavenging, calculated using the following equation:

$$\mathrm{DPPH}\;\mathrm{scavenging}\;\mathrm{activity}\;(\%)=\frac{A_{\mathrm{control}}-A_{\mathrm{sample}}}{A_{\mathrm{control}}}\times 100$$

where $A_{\mathrm{control}}$ represents the absorbance of the ascorbic acid control, and $A_{\mathrm{sample}}$ represents the absorbance of thymoquinone. The IC_50_ value, defined as the concentration required to scavenge 50% of DPPH radicals, was calculated for both thymoquinone and the standard. Percent scavenging was plotted against the logarithm of the concentration, and IC_50_ values were determined by nonlinear regression analysis using GraphPad Prism software 8.2.0. All measurements were performed in triplicate, and results are expressed as mean ± standard deviation (SD).

#### 4.6.2. ABTS Radical Cation Decolorization Assay

The ABTS radical scavenging activity of the purified thymoquinone was evaluated according to the method of Re et al. [71], with minor modifications. Briefly, the ABTS•^+^ radical cation was generated by reacting a 7 mmol/L ABTS solution with 2.4 mmol/L potassium persulfate, followed by incubation of the mixture in the dark at 25 °C for 12–16 h. The resulting ABTS•^+^ solution was subsequently diluted with ethanol (1:89, *v*/*v*) to obtain an absorbance of 0.70 ± 0.02 at 734 nm. Serial concentrations of purified thymoquinone and ascorbic acid (7.81–1000 μg/mL) were prepared and mixed with the diluted ABTS•^+^ solution, with methanol used as the blank. After incubation, absorbance was recorded at 734 nm against methanol as a blank. The percentage of radical scavenging activity was calculated relative to the control, and the results are expressed as mean ± standard deviation (SD) of three independent experiments.

### 4.7. Anti-Inflammatory Activity

The anti-inflammatory activity of the purified thymoquinone was assessed using the HRBC membrane stabilization assay, following the method described by Elbestawy [69]. Fresh human blood was obtained from a healthy volunteer who had not consumed non-steroidal anti-inflammatory drugs (NSAIDs) for at least one week prior to sample collection. All procedures involving human participants were conducted in accordance with national ethical and biosafety regulations. Ethical approval was granted under Approval H-2024-519-24. The blood was mixed in an equal volume with Alsever’s solution (2% dextrose, 0.8% sodium citrate, 0.5% citric acid, and 0.42% NaCl) and centrifuged at 3000 rpm. The erythrocyte pellet was washed three times with isotonic saline (0.9% NaCl) and resuspended in the same solution to prepare a 10% (*v*/*v*) HRBC suspension. Serial concentrations of purified thymoquinone (1, 2, 4, 8, 16, 32, 64, and 128 μg/mL) were prepared in dimethyl sulfoxide (DMSO). Sodium diclofenac at the same concentrations was used as the reference anti-inflammatory drug, and DMSO served as the control. For each assay, 1 mL of phosphate buffer, 2 mL of hypotonic saline, and 0.5 mL of the HRBC suspension were mixed with the test or control solutions. The reaction mixtures were incubated at 37 °C for 30 min, followed by centrifugation at 3000 rpm for 20 min. The hemoglobin content of the supernatant was quantified spectrophotometrically at 560 nm using isotonic saline (0.9% NaCl) as the blank (control). The percentage inhibition of hemolysis was calculated using the following equation:

$$\mathrm{Inhibition}\;\mathrm{of}\;\mathrm{hemolysis}\;(\%)=\frac{A_{\mathrm{control}}-A_{\mathrm{sample}}}{A_{\mathrm{control}}}\times 100$$

where $A_{\mathrm{control}}$ and $A_{\mathrm{sample}}$ represent the absorbance of the control and test sample, respectively. IC_50_ values were then determined by plotting percentage inhibition against the logarithm of concentration and calculating the concentration corresponding to 50% inhibition using nonlinear regression analysis (GraphPad Prism).

### 4.8. Antidiabetic Activity

#### 4.8.1. α-Amylase Inhibition Assay

The α-amylase inhibitory activity of purified thymoquinone was evaluated using a modified Saravanan method [72]. Briefly, various concentrations of purified thymoquinone (1–128 μg/mL) were pre-incubated with 500 μL of 0.02 mol/L sodium phosphate buffer (pH 6.9, containing 0.006 mol/L NaCl) and 0.5 mg/mL α-amylase from porcine pancreas (Sigma-Aldrich, Cat. No. A3176) at 25 °C for 10 min. Acarbose at the same concentrations was used as positive control. Following pre-incubation, 500 μL of 1% (*w*/*v*) soluble starch solution prepared in the same buffer was added, and the reaction was further incubated for 10 min. The enzymatic reaction was terminated by adding 1.0 mL of dinitrosalicylic acid (DNS) reagent (96 mM), followed by heating in a boiling water bath for 5 min. After cooling to room temperature, the reaction mixture was diluted with 10 mL of distilled water, and absorbance was measured at 450 nm against 1 mL buffer and 1 mL 3,5-dinitrosalicylic acid (DNS) solutions as a blank.

#### 4.8.2. α-Glucosidase Inhibition Assay

For the α-glucosidase inhibition assay, serial concentrations of purified thymoquinone or acarbose (1–128 μg/mL) were prepared and combined with 100 μL of 0.1 M phosphate buffer (pH 6.9) containing α-glucosidase (yeast, Sisco Research Laboratories Pvt. Ltd., Mumbai, India; 1.0 U/mL) in 96-well microplates, followed by incubation at 25 °C for 10 min. Subsequently, 50 μL of 5 mM *p*-nitrophenyl-α-D-glucopyranoside (pNPG; Sigma-Aldrich, Cat. No. N7006) in the same buffer was added to each well, and the reaction mixtures were incubated for an additional 5 min at 25 °C. Absorbance was measured at 405 nm before and after incubation using a microplate reader (VMax, Molecular Devices, Sunnyvale, CA, USA). Control (blank) wells contained 50 μL of buffer in place of the purified thymoquinone [73]. The α-glucosidase inhibitory activity was expressed as percent inhibition, calculated using the following equation:

$$\mathrm{Inhibition}\;(\%)=\frac{\left(A_{\mathrm{sample}}-A_{\mathrm{sample}\;\mathrm{blank}}\right)}{\left(A_{\mathrm{control}}-A_{\mathrm{control}\;\mathrm{blank}}\right)}\times 100$$

### 4.9. Statistical Analysis

The statistical tests applied, including two-way ANOVA followed by Tukey’s post hoc test, significance thresholds (*p* < 0.05), and the number of biological replicates (n = 3). Statistical annotations have been standardized across all tables and figures.

## 5. Conclusions

This study provides a comprehensive and integrative evaluation of *N. sativa* seeds by combining advanced phytochemical profiling with multi-level biological assessments, thereby addressing key gaps in previously published work. This investigation is distinguished by the systematic integration of high-resolution UHPLC/QTOF-MS based metabolite profiling with targeted purification of thymoquinone, followed by comprehensive evaluation across antibacterial, antibiofilm, antioxidant, anti-inflammatory, and antidiabetic assays. In contrast to previous studies that addressed isolated biological endpoints, the present work establishes a unified analytical-to-functional framework, thereby strengthening mechanistic insight into the therapeutic potential of *N. sativa*.

Thymoquinone was unequivocally identified as the dominant bioactive constituent and demonstrated broad-spectrum antibacterial activity, significant inhibition of biofilm formation at sub-inhibitory concentrations, strong free-radical scavenging capacity, effective membrane-stabilizing anti-inflammatory activity, and selective inhibition of α-glucosidase over α-amylase. This selective enzyme inhibition represents a pharmacologically advantageous feature, suggesting reduced gastrointestinal side effects compared with conventional antidiabetic drugs. Collectively, these findings position thymoquinone as a promising multi-target natural compound with relevance for managing infectious, inflammatory, oxidative, and metabolic disorders. Despite these strengths, certain limitations should be acknowledged. The biological activities were evaluated primarily using in vitro models, which, while informative, do not fully recapitulate in vivo pharmacokinetics, bioavailability, or metabolic transformation. Furthermore, potential synergistic interactions between thymoquinone and other minor constituents of *N. sativa* were not explored and warrant further investigation. Future studies should focus on in vivo validation of the observed bioactivity, detailed molecular docking and mechanistic analyses, and comprehensive toxicity and pharmacokinetic profiling to assess safety and therapeutic windows. Exploring formulation strategies to enhance the stability and bioavailability of thymoquinone, as well as evaluating its synergistic potential with standard antimicrobial and antidiabetic agents, may further enhance its clinical applicability. Overall, this study lays a robust scientific foundation for the continued development of *N. sativa*-derived compounds and supports thymoquinone as a viable candidate for translational and clinical research.

## Figures and Tables

**Table 1:** Quantitative phytochemical composition of *N. sativa* seed extracts.

Phytochemical	Hexane	Ethyl Acetate	Methanol	Ethanol
Saponins mg/mL	ND	1.84 ± 0.12	3.96 ± 0.21	3.45 ± 0.18
Alkaloids mg/mL	0.92 ± 0.06	2.35 ± 0.15	4.21 ± 0.24	3.88 ± 0.19
Tannins mg TAE/mL	ND	1.62 ± 0.10	3.58 ± 0.17	3.21 ± 0.14
Flavonoids mg QE/mL	0.68 ± 0.05	2.94 ± 0.16	5.12 ± 0.26	4.78 ± 0.23
Phenols mg GAE/mL	1.25 ± 0.08	3.46 ± 0.18	6.34 ± 0.31	5.89 ± 0.28
Steroids mg CE/mL	2.88 ± 0.14	2.15 ± 0.12	1.42 ± 0.09	1.76 ± 0.11
Terpenoids mg/mL	3.21 ± 0.17	2.64 ± 0.14	2.08 ± 0.12	2.31 ± 0.13

ND = Not detected.

**Table 2:** UHPLC/QTOF-MS profiling *N. sativa* seed methanolic extract.

Peak No.	Retention Time (min)	Molecular Formula	Molecular Weight	Ion Mode	Tentative Identification	Relative Abundance (%)	Compound Class
1	5.21	C_10_H_12_O	165.0912	[M + H]^+^	Thymoquinone	58	Quinone
2	14–15	C_10_H_14_	135.1176	[M + H]^+^	*p*-Cymene	13	Monoterpene
3	21.0	C_10_H_14_O	151.1123	[M + H]^+^	Carvacrol	9	Phenolic monoterpene
4	29.12	C_15_H_24_	204.36	[M + H]^+^	Longifolene	10	Sesquiterpene
5	37.32	C_18_H_18_N_2_O_2_	294.35	[M + H]^+^	Nigellidine	10	Alkaloid

**Table 3:** Antibacterial activity of thymoquinone against standard strains and foodborne bacterial strains.

Bacterial Strains	Thymoquinone		Ciprofloxacin		Statistical Comparison *p*-Value
	IZD (mm)	MIC (µg/mL)	IZD (mm)	MIC (µg/mL)	
*Klebsiella pneumoniae* ATCC 4352	15.2 ± 0.8	250	28.6 ± 1.1	0.25	<0.001
*Pseudomonas aeruginosa* ATCC 27853	13.4 ± 0.6	500	26.9 ± 1.0	0.50	<0.001
*Escherichia coli ATCC* 25922	16.1 ± 0.7	125	30.2 ± 1.3	0.06	<0.001
*Bacillus subtilis* ATCC 6633	20.5 ± 0.9	62.5	32.4 ± 1.2	0.12	<0.001
*Staphylococcus aureus* ATCC 29213	21.3 ± 1.0	62.5	33.1 ± 1.4	0.25	<0.001
*Salmonella typhimurium* ATCC 35987	14.8 ± 0.5	250	27.5 ± 1.1	0.12	<0.001
*Listeria monocytogenes*	22.6 ± 0.9	62.5	31.8 ± 1.3	0.50	<0.01
*Enterococcus faecalis*	18.9 ± 0.7	125	29.4 ± 1.2	1.00	<0.001
*Staphylococcus aureus*	19.7 ± 0.8	125	30.6 ± 1.5	0.50	<0.001
*Escherichia coli*	14.3 ± 0.6	250	27.1 ± 1.0	0.12	<0.001

IZD = inhibiting zone diameter (mm).

**Table 4:** Antioxidant activity of thymoquinone evaluated by DPPH and ABTS assays.

Concentration (µg/mL)	DPPH Scavenging (%)		ABTS Scavenging (%)	
	Thymoquinone	Ascorbic Acid	Thymoquinone	Ascorbic Acid
**7.81**	18.6 ± 1.4	32.4 ± 1.6 ^a^	22.1 ± 1.8	38.7 ± 2.0 ^a^
**15.62**	28.9 ± 1.7	45.8 ± 2.1 ^a^	34.6 ± 2.0	52.3 ± 2.4 ^a^
**31.25**	41.7 ± 2.2	61.9 ± 2.6 ^b^	48.9 ± 2.5	69.5 ± 2.8 ^b^
**62.5**	56.8 ± 2.9	74.6 ± 3.1 ^b^	63.7 ± 3.0	82.8 ± 3.3 ^b^
**125**	69.5 ± 3.4	85.9 ± 3.6 ^c^	76.8 ± 3.5	91.6 ± 3.8 ^c^
**250**	80.4 ± 3.9	92.8 ± 4.1 ^c^	88.1 ± 3.9	96.3 ± 4.0 ^c^
**500**	89.2 ± 4.2	96.7 ± 4.4 ^c^	94.5 ± 4.1	98.4 ± 4.3 ^c^
**1000**	94.6 ± 4.6	98.9 ± 4.8 ^c^	97.8 ± 4.4	99.6 ± 4.7 ^c^

^a^ *p* < 0.05 first two rows, ^b^ 0.05 < *p* < 0.001 rows 3–6 and ^c^ *p* < 0.001.

**Table 5:** Anti-inflammatory activity of thymoquinone assessed by HRBC membrane stabilization assay.

Concentration (µg/mL)	% Inhibition of Hemolysis, Mean ± SD	
	Thymoquinone	Sodium Diclofenac
**1**	12.4 ± 1.1	18.6 ± 1.3 ^a^
**2**	18.9 ± 1.4	26.2 ± 1.6 ^a^
**4**	27.5 ± 1.8	38.9 ± 2.0 ^b^
**8**	34.8 ± 2.1	47.6 ± 2.4 ^b^
**16**	48.6 ± 2.7	63.4 ± 3.1 ^b^
**32**	61.9 ± 3.3	76.8 ± 3.6 ^c^
**64**	73.2 ± 3.9	88.5 ± 4.1 ^c^
**128**	85.6 ± 4.4	94.2 ± 4.7 ^c^

^a^ *p* < 0.05 first two rows, ^b^ 0.05 < *p* < 0.001 rows 3–5 and ^c^ *p* < 0.001.

## Data Availability

The original contributions presented in this study are included in the article material. Further inquiries can be directed to the corresponding author.
